# OVERTURE Phase 2 Clinical Trial MRI Outcomes Demonstrate Consistent Treatment Benefits in Subgroup Analysis

**DOI:** 10.1002/alz.085091

**Published:** 2025-01-09

**Authors:** Ralph Kern, Colin Kuang, Chandran Seshagiri, Alexandra Konisky, Alyssa Galley, Alyssa Boasso, Evan Hempel, Zach Malchano, Brent Vaughan, Aylin Cimenser, Xiao Da, Monika Shpokayte, Mihaly Hajos

**Affiliations:** ^1^ Cognito Therapeutics, Cambridge, MA USA

## Abstract

**Background:**

The OVERTURE (NCT03556280) randomized controlled trial (RCT) evaluated evoked gamma oscillation therapy using Cognito’s medical device (CogTx‐001) in mild‐moderate Alzheimer’s disease (AD). Brain atrophy, as measured by whole brain volume (WBV), is a key indicator of neurodegeneration in AD^1^ and may be influenced by baseline covariates such as age^2^, disease stage^3^, APOE4 carrier status^4^, and PET amyloid levels^5^, as well as treatment^6^. We analyzed OVERTURE MRI whole brain volume outcomes across these key baseline subgroups.

**Method:**

Neuroimaging data were collected in the Overture phase 2 clinical trial (NCT03555280), comparing one‐hour, daily, at‐home, 40Hz gamma sensory stimulation vs. sham stimulation for six months (2:1 randomization). 76 participants were randomized, 74 were treated and 53 completed the 6‐month RCT. Magnetic resonance imaging (1.5 T‐MRI and FLAIR) data were collected at baseline (N=56) and month 6 (N=53). Brain amyloid was evaluated with [18F]‐florbetapir PET at baseline and 6 months. APOE4 carrier status was evaluated at baseline.

**Result:**

Baseline WBV (mean, SD) in active (1060.7,129.35) and sham‐treated participants (1017.7,122.80) and baseline PET SUVR in active (1.3,0.23) and sham‐treated participants (1.2,0.25) were similar. We observed a 69% reduction in MRI whole brain volume loss in participants who received active treatment compared to sham treatment (p=0.005). Treatment outcomes were similar across subgroups: baseline age (>70, ≤70), Mild vs. moderate AD (MMSE 20‐26, 14‐19), APOE4 genotype (carrier, non‐carrier), and PET amyloid values (SUVR ≥1.12, <1.12).

**Conclusion:**

The OVERTURE clinical trial demonstrated consistent treatment‐related slowing of brain atrophy across key baseline subgroups (age, AD stage, APOE4 carrier status, and PET amyloid). We are evaluating WBV as a key indicator of neurodegeneration in the ongoing phase 3 HOPE study (NCT05637801).

**References**:

Jack, Alzheimer Dement. 2018

Coupé, Sci Rep. 2019

Wei X, Cereb Cortex 2023

Löwe, PLOSOne 2016

La Joie, Sci Transl Med. 2020

Ten Kate M et al. J Prev Alz Dis 2023;92

